# Human amnion mesenchymal cells inhibit lipopolysaccharide-induced TNF-α and IL-1β production in THP-1 cells

**DOI:** 10.1186/s40659-015-0062-3

**Published:** 2015-12-23

**Authors:** Jun Shu, Xiaojuan He, Lan Zhang, Hong Li, Ping Wang, Xiaojie Huang

**Affiliations:** Institute of Clinical Medical Science, China-Japan Friendship Hospital, 100029 Beijing, China; Institute of Basic Research in Clinical Medicine, China Academy of Chinese Medical Sciences, 100700 Beijing, China

**Keywords:** Human amnion mesenchymal cells, THP-1 cells, TNF-α, IL-1β, Immunosuppression

## Abstract

**Background:**

Human amnion mesenchymal cells (hAMCs), isolated from the amniotic membrane of human placenta, are a unique population of mesenchymal stem cells. Recent studies demonstrated that hAMCs could inhibit the activities and functions of several immune cells. However, their effect on inflammatory macrophages is largely unknown. This study investigated the effect of hAMCs on expression of inflammatory cytokines and mitogen-activated protein kinases (MAPKs)/NF-κB pathway in human THP-1 macrophages induced by lipopolysaccharide (LPS).

**Results:**

The levels of TNF-α and IL-1β secreted by LPS- stimulated THP-1 cells were increased significantly compared with those in the control group. After co-culture with different numbers of hAMCs, the levels of TNF-α and IL-1β in LPS-stimulated THP-1 cells were significantly reduced compared with the LPS group. The mRNA expression of TNF-α and IL-1β were also markedly inhibited. Moreover, treating LPS-stimulated THP-1 cells with hAMCs supernatants could also suppress TNF-α and IL-1β production in THP-1 cells. Important signaling pathways involved in the production of TNF-α and IL-1β were affected by hAMCs co-culture: hAMCs remarkably suppressed NF-κB activation and down-regulated the phosphorylation of ERK and JNK in LPS- stimulated THP-1 cells.

**Conclusions:**

Human amnion mesenchymal cells inhibited the production of TNF-α and IL-1β secreted by LPS-stimulated THP-1 cells, partly through the suppression of NF-κB activation and ERK and JNK phosphorylation.

## Background

Mesenchymal stem cells (MSCs), which have been successfully isolated from bone marrow, adipose tissue, umbilical cord blood, amniotic fluid, and peripheral blood, amongst other tissues, are multipotent cells that can differentiate into a variety of cell types, including osteoblasts, chondrocytes and adipocytes [1]. Recent studies have demonstrated that MSCs possessed immunosuppressive and immunoregulatory activities [2]. Mesenchymal stem cells have been successfully used in the treatment of graft-versus-host disease and some autoimmune diseases such as insulin-dependent diabetes mellitus, experimental autoimmune encephalomyelitis and rheumatoid arthritis [3–6].

Human amnion mesenchymal cells (hAMCs) are isolated from the amniotic membrane of human placenta. These cells possess stem cell characteristics and differentiation potential [7]. Because hAMCs have a number of advantages including easily obtained, relatively exempt from ethical problem, do not express telomerase, and have a low risk of tumor formation, they might represent a new ideal MSCs resource for clinical application [8]. Recent studies have indicated that hAMCs also had immunomodulatory functions, including influencing T cell proliferation, and inhibiting dendritic cell (DC) differentiation and maturation [9, 10]. However, whether hAMCs may regulate the activities of macrophages is still unknown.

Inflammation plays an important role in the progression of many diseases, including cancer and autoimmune diseases [11, 12]. Macrophages are regarded as the key inflammatory cells associated with the pathologic process of inflammation [13]. Studies commonly investigate the response of THP-1 cells, an immortalized human monocyte/macrophage cell line, to lipopolysaccharide (LPS) challenge as an appropriate cell model system to study macrophage activation [14, 15]. It was reported that LPS elicited the expression of multiple pro-inflammatory cytokines such as TNF-α and IL-1β in THP-1 cells, partly through the mitogen-activated protein kinase (MAPK)/NF-κB signaling pathway [16, 17]. Therefore, this study investigated the effect of hAMCs on the production of inflammatory cytokines and the regulation of the MAPK/NF-κB signaling pathway in LPS-stimulated THP-1 cells, a classic inflammatory macrophage model.

## Results

### Morphological characterization of isolated hAMCs

**Fig. 1:**
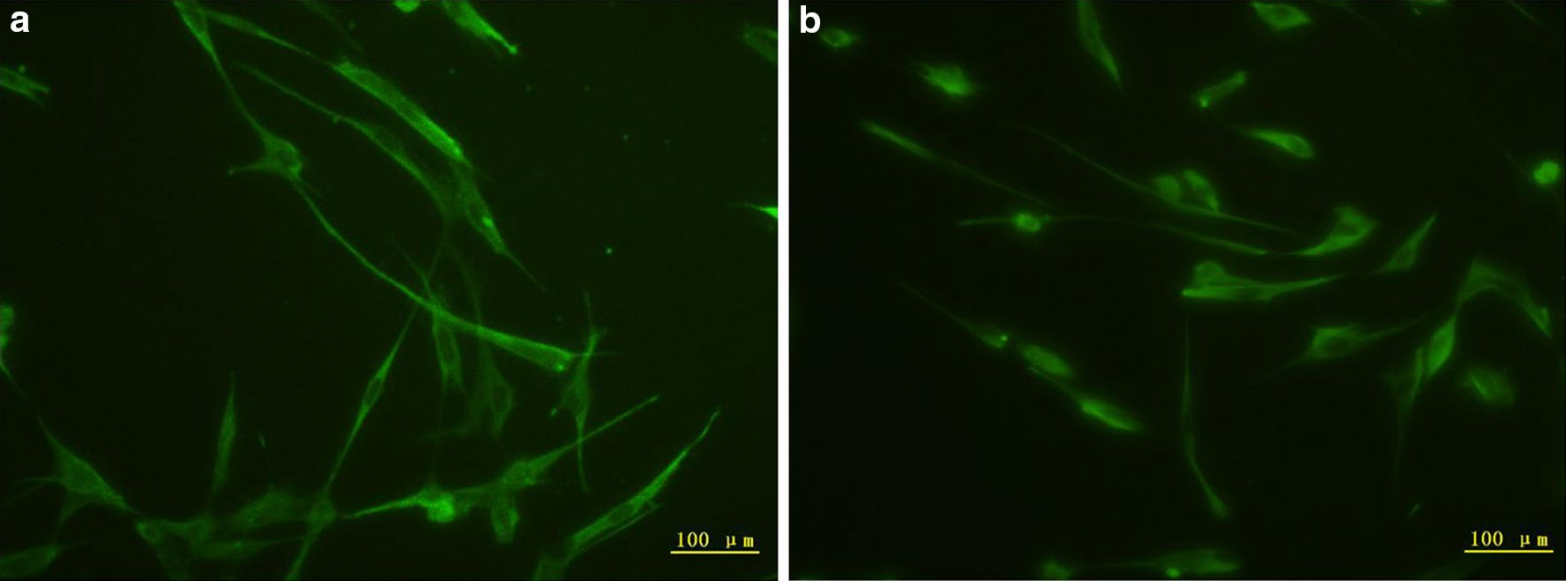
hAMCs express mesenchymal stem cell specific marker STRO-1 and vimentin. hAMCs were seeded onto 24-well plates and fixed by 4 % paraformaldehyde. After being blocked, cells were incubated with a mouse anti-human vimentin antibody or a mouse anti-human STRO-1 antibody respectively overnight at 4 °C. After being incubated with fluorescence-conjugated secondary antibodies, the cells were observed under a fluorescent microscope. **a** Expression of mesenchymal marker STRO-1 in hAMCs. **b** Expression of mesenchymal marker vimentin in hAMCs. Magnification: ×100

Human amnion mesenchymal cells presented a colony-like growth, with a mostly oval, spindle or polygonal in shape, and exhibiting a typical mesenchymal morphology. To identify hAMCs further, we performed immunofluorescence staining with antibodies to STRO-1 and vimentin, two mesenchymal stem cell specific markers. The results showed that these cells expressed STRO-1 and vimentin in the cell cytoplasm (Fig. 1).

### hAMCs co-culture inhibit TNF-α and IL-1β production in LPS-stimulated THP-1 cells

**Fig. 2:**
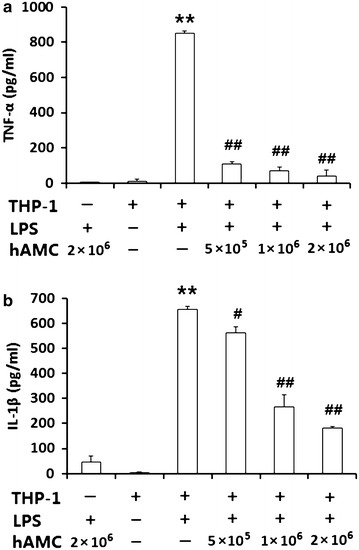
hAMCs co-culture inhibits LPS-induced TNF-α and IL-1β production in THP-1 cells. THP-1 cells were pre-treated with PMA to induce macrophage. Then, they were stimulated with 10 μg/mL LPS with or without different numbers of hAMCs (5 × 10^5^, 1 × 10^6^, 2 × 10^6^) in a 12-well transwell plate for 24 h at 37 °C. Expression levels of TNF-α (**a**) and IL-1β (**b**) in the culture supernatants were detected by ELISA. Data represent the mean ± S.D. of three independent experiments. **P < 0.01, vs. THP-1(+) LPS (−) group; ^#^P < 0.05, ^##^P < 0.01, vs. THP-1(+) LPS (+) group

To evaluate the effect of hAMCs on the expression of pro-inflammatory cytokines in LPS-stimulated THP-1 cells, we measured the levels of TNF-α and IL-1β, two classic pro-inflammatory cytokines. As shown in Fig. 2, hAMCs secreted low concentrations of TNF-α (4.24 ± 0.89 pg/mL) and IL-1β (47.47 ± 23.14 pg/mL) when treated with LPS for 24 h. Compared with the negative control group, TNF-α and IL-1β production in THP-1 cells was remarkably increased after LPS stimulation (TNF-α = 849.36 ± 13.94 pg/mL and IL-1β = 655.98 ± 10.25 pg/mL). After co-culture with hAMCs for 24 h, TNF-α and IL-1β levels in LSP-stimulated THP-1 cells was significantly decreased. The inhibitory effect was concentration-dependent. When the ratio of hAMCs:THP-1 was 2:1, the TNF-α and IL-1β levels in supernatant were decreased to 39.76 ± 33.41 pg/mL and 182.82 ± 3.68 pg/mL, respectively.

### hAMCs co-culture inhibit TNF-α and IL-1β mRNA expression in LPS-stimulated THP-1 cells

**Fig. 3:**
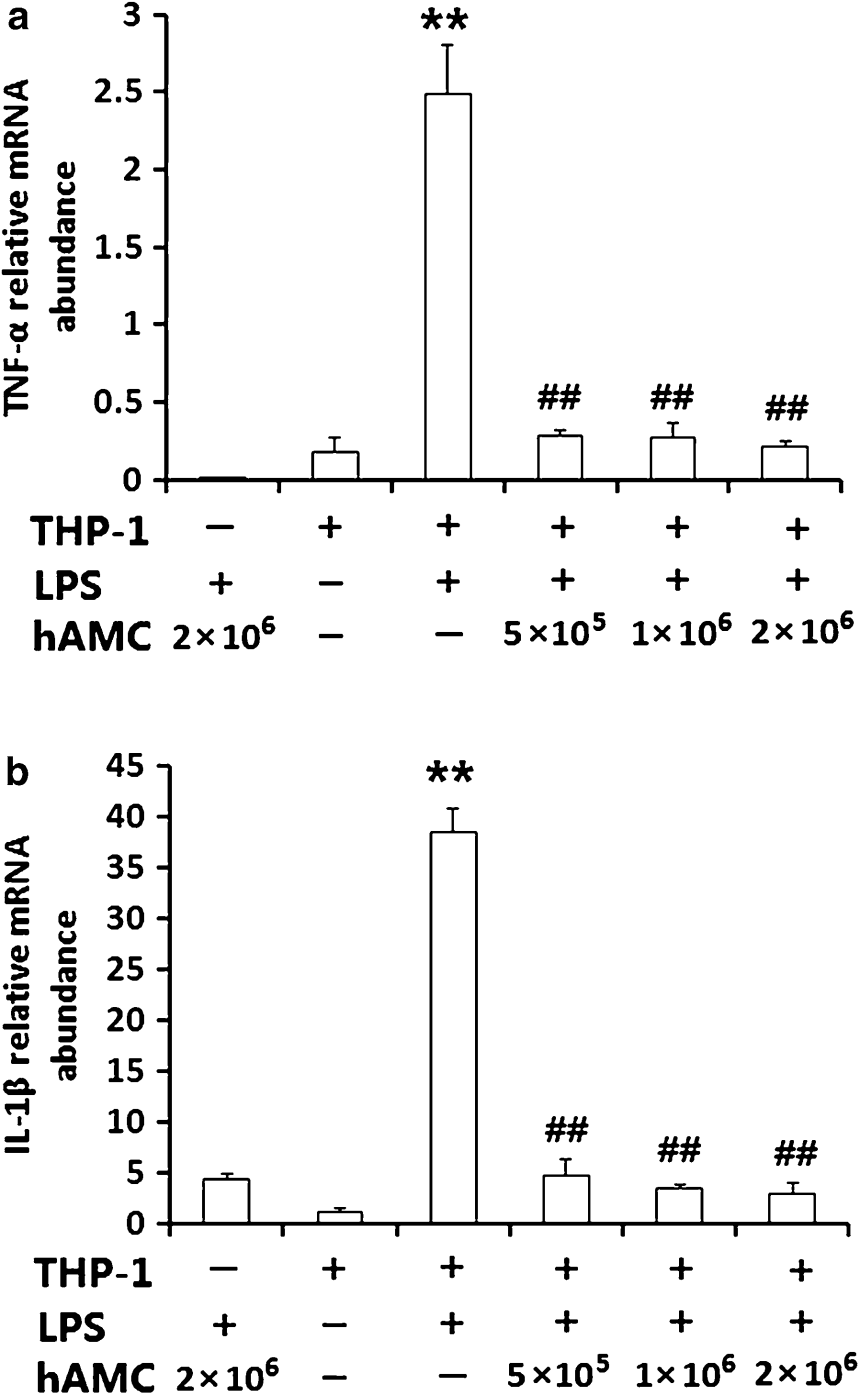
hAMCs co-culture inhibits the mRNA expression of TNF-α and IL-1β in LPS-stimulated THP-1 cells. THP-1 cells were pre-treated with PMA to induce macrophage. Then, they were stimulated with 10 μg/mL LPS with or without different numbers of hAMCs (5 × 10^5^, 1 × 10^6^, 2 × 10^6^) in a 12-well transwell plate for 24 h at 37 °C. TNF-α mRNA (**a**) and IL-1β mRNA (**b**) expressions were determined by real time-PCR. GAPDH was used as a quantitative control. All experiments were performed three times. **P < 0.01, vs. THP-1(+) LPS (−) group; ^##^P < 0.01, vs. THP-1(+) LPS (+) group

To detect changes in TNF-α and IL-1β levels in LPS-stimulated THP-1 cells further, real-time PCR was used to measure cytokine mRNA expression. As shown in Fig. 3, the relative mRNA expression of TNF-α (0.007 ± 0.002) and IL-1β (4.408 ± 0.41) were low in hAMCs when treated with LPS for 24 h. Compared with the negative control group, the relative TNF-α and IL-1β mRNA expression in THP-1 cells was remarkably increased after stimulation with LPS, reaching 2.483 ± 0.323 and 38.5 ± 2.323, respectively. After co-culture with hAMCs for 24 h, the relative TNF-α and IL-1β mRNA expression in LSP-stimulated THP-1 cells was significantly decreased. The inhibitory effect was concentration-dependent. When the ratio of hAMCs: THP-1 was 2:1, the relative TNF-α and IL-1β mRNA expression in LPS-stimulated THP-1 cells was decreased to 0.214 ± 0.032 and 2.928 ± 0.955, respectively.

### hAMCs supernatants inhibit TNF-α and IL-1β production in LPS-stimulated THP-1 cells

**Fig. 4:**
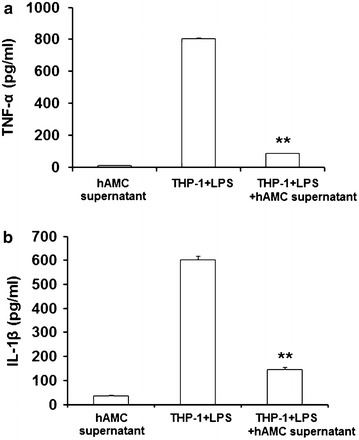
hAMCs supernatants inhibit LPS-induced TNF-α and IL-1β production in THP-1 cells. THP-1 cells were pre-treated with PMA to induce macrophage. Then, they were stimulated with LPS (10 μg/mL) with or without hAMCs supernatants for 24 h at 37 °C. Expression levels of TNF-α (**a**) and IL-1β (**b**) in the culture supernatants of THP-1 cells were detected by ELISA. Data represent the mean ± S.D. of three independent experiments. **P < 0.01, vs. THP-1 + LPS group

To further confirm the effect of hAMCs, we collected the supernatants of hAMCs and added them into LPS-stimulated THP-1 cells, and the same results were found. As shown in Fig. 4, hAMCs supernatants also significantly inhibited the production of pro-inflammatory cytokines TNF-α and IL-1β in LPS-stimulated THP-1 cells (P < 0.01).

### hAMCs have no inhibitory effect on THP-1 cells viability

**Fig. 5:**
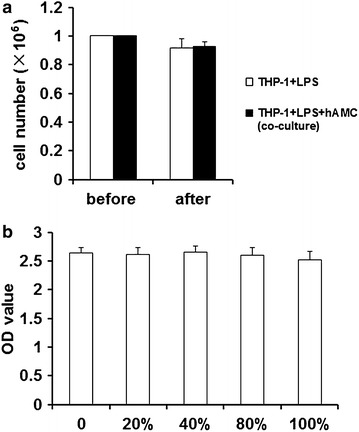
hAMCs have no inhibitory effect on THP-1 cells viability. **a** Cells counting result. PMA-induced THP-1 cells were treated with LPS (10 μg/mL) with or without hAMCs in a 12-well transwell plate for 24 h at 37 °C. The THP-1 cells were then collected and counted. **b** CCK-8 assay result. THP-1 cells were firstly induced by PMA for 48 h. Then, different concentrations of hAMCs supernatants (0, 20, 40, 80, and 100 %) were added. 20 h later, 10 μL of CCK-8 reagent was added into each well and the cells were incubated for another 4 h. Finally, the absorbance of each well was measured at 450 nm. All experiments were performed three times. Data are the mean ± S.D. of three independent experiments

To investigate whether hAMCs supernatant or co-culture had inhibitory effect on THP-1 cells viability, we perform CCK-8 assay and cell counting. As shown in Fig. 5, 20–100 % hAMCs supernatants did not display any cellular toxicity against LPS-stimulated THP-1 cells over 24 h. Further, co-culture of hAMCs with LPS-stimulated THP-1 cells for 24 h did not change the cell number of THP-1 cells. Before co-culture, 1 × 10^6^ THP-1 cells were added into the wells of a transwell plate. After co-culture for 24 h, there was no significantly difference between THP-1+LPS group and THP-1+LPS+hAMCs (co-culture) group. The THP-1 cells numbers in two groups were 0.92 × 10^6^ and 0.93 × 10^6^, respectively.

### hAMCs suppress the phosphorylation of ERK and JNK in LPS-stimulated THP-1 cells

**Fig. 6:**
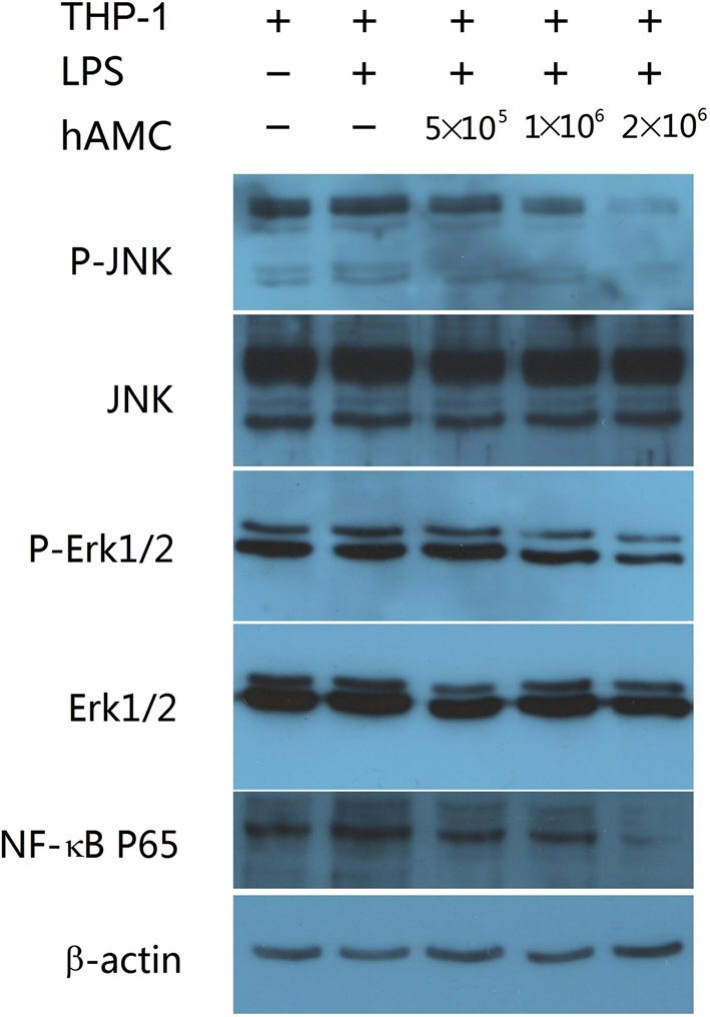
hAMCs inhibit the phosphorylation of MAPKs and activation of NF-κB in LPS-stimulated THP-1 cells. PMA-induced THP-1 cells were treated with LPS (10 μg/mL) with or without hAMCs (5 × 10^5^, 1 × 10^6^, 2 × 10^6^) in a 12-well transwell plate for 24 h at 37 °C. The cells were then collected and extracted. Western blot analysis was performed to detect p-ERK, total ERK, p-JNK, total JNK and p65. β-actin was used as an internal control

To determine whether MAPK signaling pathways were involved in the regulatory effects of hAMCs on THP-1 cells, we investigated the phosphorylation of two MAPK signaling molecules, ERK1/2 and JNK, and the activation of NF-κB. Western blot results showed that the levels of phosphorylated ERK1/2 and JNK, and NF-κB p65 were significantly up-regulated in THP-1 cells stimulated with LPS, whereas their levels were remarkably decreased in LPS-stimulated THP-1 cells after co-culture with hAMCs for 24 h. However, the total levels of ERK and JNK did not significantly change among these groups (Fig. 6).

## Discussion

Recently, MSCs have received wide attention for their differentiation potential and immunomodulatory properties in regenerative medicine. Like MSCs from other tissues, hAMCs also have immuno-inhibitory activities. A few groups have investigated hAMCs’ potential for treating immune-related diseases, such as multiple sclerosis, liver fibrosis, and spinal cord injury [18–20]. Although these studies demonstrate the anti-inflammatory effect of hAMCs, their mechanism of action is still poorly understood. In this study, we demonstrated that hAMCs reduced the production of TNF-α and IL-1β secreted by LPS-stimulated THP-1 cells, a model of inflammatory cell activation, which might be partly through suppressing the MAPK/NF-κB signal pathway.

Inflammation is the first response to infection and injury and is critical to body defense, and restores and maintains homeostasis. However, excessive inflammation can cause inflammatory diseases [21]. Macrophages are regarded as a major component in the inflammatory response [22, 23]. When stimulated by bacterial endotoxin, e.g. LPS, macrophages can secrete large amounts of inflammatory cytokines, such as TNF-α and IL-1β, and these inflammatory cytokines further activate macrophages, causing a cascade of inflammatory response [15]. In this study, we co-cultured hAMCs with LPS-stimulated THP-1 cells and found that hAMCs effectively inhibited pro-inflammatory cytokine production in THP-1 cells. Moreover, treating LPS-stimulated THP-1 cells with hAMCs supernatants could also suppress TNF-α and IL-1β production in THP-1 cells. On the other hand, supernatants of LPS-stimulated THP-1 cells could not promote hAMCs secreting TNF-α and IL-1β (data not shown). Further, hAMCs supernatants did not display any cellular toxicity against LPS-stimulated THP-1 cells, which could exclude a nonspecific cytotoxicity as a possible explanation for the decreased cytokines output. Therefore, all these results further supported the anti-inflammatory activity of hAMCs.

Mitogen-activated protein kinases are a family of serine/threonine protein kinases, including ERKs and JNKs, which are regulated by a phosphorylation cascade, with a prototype of three protein kinases that sequentially phosphorylate one another [24]. Mitogen-activated protein kinases are responsible for most cellular responses to cytokines and are crucial for regulation of the production of inflammatory mediators [25]. To investigate whether these pathways were involved in the molecular mechanisms of the suppressive effect of hAMCs on LPS-stimulated THP-1 cells, we measured the levels of ERK and JNK phosphorylation. The levels of phosphorylation of ERK and JNK were up-regulated in THP-1 cells induced by LPS, but were significantly down-regulated after co-culture with hAMCs. These results indicated that hAMCs inhibited TNF-α and IL-1β production in macrophages by regulating MAPKs signaling pathways.

NF-κB is a major transcription factor that regulates both innate and adaptive immune responses. After being activated by a variety of stimuli, including LPS, it translocates to the nucleus and binds its cognate DNA binding site to regulate the transcription of numerous genes, including cytokines, chemokines and stress-response proteins. NF-κB is a pivotal regulator of pro-inflammatory gene expression and is thought to be associated with some inflammatory diseases [26] because it is highly activated at sites of inflammation and the administration of NF-kB decoys is effective in an animal model of rheumatoid arthritis [27]. In the present experiment, co-culture with hAMCs remarkably down-regulated NF-κB level in LPS-stimulated THP-1 cells, which demonstrated that the NF-κB signaling pathway was associated with the inhibitory effect of hAMCs on THP-1 cells. These results were also in accordance with previous reports. Co-culture of bone marrow-derived MSCs with macrophages down-regulated COX-2/PGE2 expression of macrophages by inhibiting the activation-associated phosphorylation of p38 MAPK and ERK [28]. Fang et al. also reported that adipose-derived MSCs transplantation suppressed inflammatory responses in rats with diabetic nephropathy induced by streptozotocin, partly by decreasing the expression of p-p38, p-ERK and p-JNK [29].

In conclusion, this study indicated that mediators secreted by hAMCs could inhibit the production of pro-inflammatory cytokines in LPS-stimulated THP-1 cells, whether in co-culture condition or just with hAMCs supernatants. However, which mediators secreted by hAMCs participated in the immuno-inhibitory effect is still unknown. Previous studies reported that several soluble factors including transforming growth factor (TGF-β), hepatocyte growth factor (HGF), prostaglandin E2 (PGE2), indoleamine 2, 3 dioxygenase (IDO) secreted by hAMCs were associated with immunomodulatory effects [30]. However, whether these factors are associated with hAMCs’ effects on LPS-stimulated THP-1 cells requires further study.

## Conclusions

Taken together, this study demonstrated that hAMCs might prevent inflammatory responses by inhibiting the activation of NF-κB and the phosphorylation of MAPK pathways in LPS-stimulated THP-1 macrophages. These findings suggest that hAMCs may be useful for the treatment of some inflammatory diseases.

## Methods

### Cell culture

THP-1, human monocyte-like cells, were purchased from Cell Culture Center of Chinese Academy of Medical Sciences and cultured in RPMI1640 medium (Gibco BRL, Life Technologies, Grand Island, NY, USA) supplemented with 10 % fetal bovine serum (FBS), 100 U/mL penicillin, and 100 µg/mL streptomycin.

### Isolation of human amnion mesenchymal cells

Human amnion mesenchymal cells were isolated from abandoned human placentas by trypsin and collagenase V according to our previous report with some modifications [8]. In brief, amnion layer was mechanically peeled off from the chorion layer and washed several times with Hanks’ balanced salt solution (HBSS) without calcium and magnesium to remove blood. Then, the amnion was digested with 0.25 % trypsin at 37 °C for 30 min. Further, the amnion was cut into pieces of 1 cm^2^ and then digested with 0.1 % collagenase V (Sigma-Aldrich, St. Louis, MO, USA) at 37 °C for 30 min followed by centrifugation (1500 rpm, 10 min) and thorough washing with phosphate buffer saline (PBS). Finally, isolated hAMCs were cultured in DMEM/F12 supplemented with 10 % FBS. hAMCs of no more than three passages were used for all experiments. All the correlated ethical issues concerning this study were approved by the corresponding institutional review board (IRB number: CJFH001035).

### hAMCs—THP-1 cell co-culture

THP-1 cells were pre-treated for 48 h with 10 ng/mL phorbol 12-myristate 13-acetate (PMA) (Sigma-Aldrich, St. Louis, MO, USA) to induce differentiation to macrophages [31]. After washing with PBS three times to remove PMA, they were stimulated with 10 μg/mL LPS (Sigma-Aldrich, St. Louis, MO, USA) with or without hAMCs in a 12-well transwell plate (0.4 μM pore size, Corning, Lowell, MA, USA) for 24 h at 37 °C. Experiments were performed with different hAMCs numbers (5 × 10^5^, 1 × 10^6^, 2 × 10^6^) and with a constant number of THP-1 cells (1 × 10^6^), to obtain ratios of hAMCs:THP-1 of 0.5:1, 1:1, and 2:1. hAMCs were cultured in the upper layer, while THP-1 cells were placed in the lower layer in RPMI1640 medium supplemented with 10 % FBS. After co-culture for 24 h, the supernatants were collected for ELISA assay, and the cells were collected and counted for real-time PCR and western blot analysis.

### THP-1 cells treatment

To further confirm the effect of hAMCs, we did a single-culture experiment. THP-1 cells were pre-treated with PMA to induce macrophage. Then, they were stimulated with LPS (10 μg/mL) with or without hAMCs supernatants (100 %) for 24 h at 37 °C. Cells culture supernatants were collected and used for ELISA assay.

### Immunofluorescence staining assay

Isolated hAMCs were seeded onto 24-well plates. After adherence, cells were washed three times with PBS and fixed by 4 % paraformaldehyde at room temperature for 30 min. Then, they were blocked with 1 % BSA for another 30 min. Subsequently, cells were incubated overnight at 4 °C with a mouse anti-human vimentin antibody (1:500 dilution, Chemicon, USA) or a mouse anti-human STRO-1 antibody (1:500 dilution, Chemicon, USA) respectively. After three washes, cells were incubated with Alexa Fluor 488-conjugated goat anti-mouse IgG (Invitrogen, Life Technologies, Grand Island, NY, USA) or FITC-conjugated goat anti-mouse IgM (Sigma-Aldrich, St. Louis, MO, USA) for 1 h. Finally, cells were observed under a fluorescent microscope.

### CCK-8 assay

THP-1 cells (1 × 10^5^ cells/mL) were seeded in 96-well plate and induced by PMA for 48 h in a 37 °C. Then, different concentrations of hAMCs supernatants were added. 20 h later, 10 μL of CCK-8 reagent was added into each well and the cells were incubated for another 4 h. Finally, the absorbance of each well was measured at 450 nm.

### ELISA assay

For co-culture experiment, the supernatants were collected after co-culture for 24 h. For single-culture experiment, the supernatants were collected after hAMCs supernatant was added into the wells of LPS-stimulated THP-1 cells for 24 h. The concentration of IL-1β and TNF-α in the culture supernatant was measured by human IL-1β ELISA kit and human TNF-α ELISA kit (eBioscience, San Diego, CA, USA), according to the manufacturer’s instructions.

### Real-time PCR analysis

To quantify the mRNA expression of IL-1β and TNF-α, real-time PCR amplification was conducted. Cells were collected and total RNA was isolated by Trizol reagent (Promega, Madison, WI, USA). The primers used were as follows: IL-1β, sense, 5′-TACAAGGAGAAGAAAGTAATGACAA-3′,antisense,5′-AGCTTGTTATTGATTTCTATCTTGT-3′; TNF-α,sense,5′-CTCCTCACCCACACCATCAGCCGCA-3′,antisense,5′-ATAGATGGGCTCATACCAGGGCTTG-3′; GAPDH, sense, 5′-CTCATGACCACAGTCCATGC-3′,antisense,5′-CACATTGGGGGTAGGAACAC-3′. GAPDH used as a quantitative control. The PCR conditions were 95 °C for 10 min, followed by 40 cycles of 95 °C for 15 s and 60 °C for 60 s. All samples were measured in triplicate. Differences in gene expression were calculated using the 2^−∆∆ct^ method.

### Western blot analysis

After being induced by PMA and co-cultured with LPS and hAMCs for 24 h at 37 °C, THP-1 cells were collected to detect the expression of ERK, JNK, and NF-κB (p65). Whole-cell lysates were prepared using ice-cold cell lysis buffer. Protein concentration was determined using a BCA protein assay kit. Samples of cell lysates were separated by 10 % SDS-PAGE and then transferred onto polyvinylidene difluoride membranes and then immuno-blotted with primary antibodies that recognized ERK (1:4000 dilution), JNK (1:2000 dilution), p-ERK (1:2000 dilution), p-JNK (1:2000 dilution), GAPDH (1:5000 dilution) and p65 (1:200 dilution) (Cell Signaling Technology, Danvers, MA, USA). Next, peroxidase conjugated secondary antibodies and ECL detection systems were used according to routine methods. β-actin protein was used as an internal control.

### Statistical analysis

All experiments were performed at least three times. Data were presented as mean ± S.D. Differences were evaluated using Statistical Package for Social Science 11.0 (SPSS18.0). Statistical analysis was performed using analysis of variance (ANOVA) and comparison of means (Student’s t test). P < 0.05 was considered statistically significant.
